# Endometrial Cancer Recurrence Risk Following Robotic Hysterectomy

**DOI:** 10.3390/curroncol33060317

**Published:** 2026-05-28

**Authors:** Sean Zhu, Ericka Wiebe, Haley Frerichs, Sunita Ghosh, Jasmine Gill, Zainab Al Habsi, Ananya Beruar, Sophia Pin

**Affiliations:** 1 Division of Gynecologic Oncology, Department of Obstetrics and Gynecology, Cumming School of Medicine, University of Calgary, Foothills Medical Centre, 1403 29 Street NW, Calgary, AB T2N 2T9, Canada; 2 Division of Radiation Oncology, Department of Oncology, Faculty of Medicine and Dentistry, University of Alberta, Cross Cancer Institute, 11560 University Avenue NW, Edmonton, AB T6G 1Z2, Canada; ericka.wiebe@albertahealthservices.ca (E.W.); zainab.alhabsi@albertahealthservices.ca (Z.A.H.); ananya.beruar@albertahealthservices.ca (A.B.); 3 Faculty of Medicine and Dentistry, University of Alberta, Walter C. Mackenzie Health Sciences Centre, 8440 112 Street NW, Edmonton, AB T6G 2R7, Canadasghosh1@ualberta.ca (S.G.); jasmine.gill@albertahealthservices.ca (J.G.); 4 Division of Gynecologic Oncology, Department of Obstetrics and Gynecology, Faculty of Medicine and Dentistry, University of Alberta, Cross Cancer Institute, 11560 University Avenue NW, Edmonton, AB T6G 1Z2, Canada; sophia.pin@albertahealthservices.ca

**Keywords:** endometrial cancer, robotic hysterectomy, recurrence, uterine manipulator, lymphovascular space invasion

## Abstract

Background: Minimally invasive surgery is widely used in endometrial cancer management, but recurrence remains a concern. Methods: A retrospective review of 1201 patients treated from 2012 to 2019 with robotic laparoscopy was conducted. Recurrence-free survival and hazard ratios were calculated using multivariate Cox models. Results: Of 1278 patients, 155 cases of recurrent disease were identified. Age (HR 1.01, *p* = 0.045), LVSI (HR 3.54, *p* < 0.001), stage and high-grade histology (HR 3.39, *p* < 0.001) were associated with increased risk of recurrence. Hazard ratios for stages IB, II and III/IV were 1.92 (*p* = 0.007), 2.67 (*p* = 0.001), and 3.23 (*p* < 0.001) respectively. The use of a uterine manipulator was an independent risk factor on multivariate analysis (HR 2.12, *p* < 0.001). Adjuvant chemotherapy (HR 0.67, *p* = 0.239) and radiotherapy (HR 0.64, *p* = 0.056) trends favored reduced risk but were not significant. Chemoradiotherapy was found to have a reduction in recurrence with a HR 0.44 (*p* = 0.002). Conclusions: Traditional prognostic factors remain important for patients with endometrial cancer having undergone robotic laparoscopic hysterectomy. Uterine manipulator use should be carefully reconsidered in endometrial cancer surgery.

## Simple Summary

This study reviews recurrence risk in patients with endometrial cancer who underwent robotic-assisted hysterectomy. Using a large single-center cohort, we evaluated clinical, pathologic, and surgical factors associated with recurrence. In addition to confirming known predictors such as stage, histology, and lymphovascular space invasion, we identified uterine manipulator use as an independent and potentially modifiable risk factor. These findings may influence surgical decision making and guide adjuvant therapy for patients at increased risk.

## 1. Introduction

Endometrial cancer is the most common gynecologic malignancy in North America and the sixth most common cancer among women globally. Its incidence has increased substantially from 1990 to 2019, especially in high-sociodemographic-index countries, likely attributable to a rising prevalence of obesity and an aging population [1,2]. Adequate techniques exist for detecting and treating early-stage disease, resulting in a five-year survival approaching 95% for appropriately treated stage I endometrioid carcinoma [3,4]. However, there is a subset of patients for whom the disease recurs despite treatment. Identifying risk factors for recurrence is crucial for guiding treatment decisions and improving morbidity and mortality.

The standard treatment for apparent early-stage endometrial cancer is total hysterectomy with bilateral salpingo-oophorectomy [5]. Most patients will also undergo surgical lymph node staging, with sentinel lymph node biopsy being diagnostically equivalent to lymphadenectomy but with a lower risk of lymphedema and postoperative complications [6,7]. Minimally invasive surgery is the preferred approach and robot-assisted techniques further reduce intraoperative complications and length of hospital stay [8]. However, surgical methods may impact recurrence risk.

Although recurrence rates after surgery for early-stage endometrial cancer are low overall, some studies suggest a small increase in risk with minimally invasive versus open surgery [9,10]. This increased risk is more evident in cervical cancer, where the Laparoscopic Approach to Cervical Cancer (LACC) trial found that minimally invasive surgery was associated with higher rates of cancer recurrence and cancer-specific death compared to open surgery [11]. One proposed explanation is that uterine manipulators used in minimally invasive procedures may disrupt tumor cells, resulting in dissemination [12]. The impact of uterine manipulators on endometrial cancer recurrence risk is controversial and may depend on the type of manipulator and patient population [13]. However, existing studies have largely evaluated heterogeneous minimally invasive cohorts, and the specific impact of these factors within exclusively robot-assisted surgical populations remains poorly defined.

After surgical treatment and staging, adjuvant therapy is considered based on the presumed risk of recurrence. Patients without residual disease or metastasis are categorized into low, intermediate, high-intermediate, and high-risk groups based on histology, stage, grade, depth of myometrial invasion, lymphovascular space invasion (LVSI) status, and age [4]. More recently, molecular classification (including POLE-mutated, mismatch repair deficient, p53-abnormal, and no specific molecular profile subtypes) has emerged as a critical component of risk stratification, refining prognostication beyond traditional clinicopathologic features and increasingly informing adjuvant treatment decisions [5]. Adjuvant radiotherapy improves locoregional disease control in the intermediate-risk group [14,15], while chemoradiotherapy is most beneficial for those in the high-risk category [16]. Patients considered low risk typically do not receive adjuvant treatment but are monitored through follow-up and routine surveillance for up to five years [17]. Although these risk groups help predict recurrence, there exists an additional layer of heterogeneity within each group which may result in undertreatment.

The objective of this study was to retrospectively review cases of endometrial cancer recurrence in patients treated with robot-assisted laparoscopic hysterectomy at a single center. By focusing on a uniform robotic surgical cohort, this study seeks to address an important gap in the literature regarding recurrence patterns and risk factors in this increasingly utilized surgical approach. We aimed to identify patient, surgical, and disease factors impacting recurrence rates to help advance guidelines for adjuvant treatment and surveillance.

## 2. Materials and Methods

### 2.1. Study Population

Ethics approval was granted through the Health Research Ethics Board of the Alberta Cancer Committee (Institutional Review Board Approval Number: HREBA.CC-21-0006_MOD1). A pre-existing database at the Royal Alexandra Hospital in Edmonton, Alberta, from January 2012 to December 2019, which included 1278 patients diagnosed with endometrial cancer, was used to screen for evidence of disease recurrence until 30 November 2023. This extended follow-up period was selected to allow for adequate capture of both early and late recurrences, given that endometrial cancer can recur several years after primary treatment, particularly in higher-risk subgroups. Cases were included in the analysis if the primary treatment was robotic-assisted laparoscopic hysterectomy with a final pathologic diagnosis of endometrial cancer. Patients were excluded if there was conversion to laparotomy, presence of unresectable disease at the time of surgery, concurrent non-endometrial cancer, or death following surgery. All tumors were staged using the 2009 FIGO staging system.

All charts were reviewed independently by four reviewers, until the cutoff date of 30 November 2023, for evidence of recurrent disease confirmed clinically or on imaging. Recurrence-free survival was calculated for the whole study population, and time to recurrence was defined as the time from primary staging surgery to recurrence.

Risk factors for analysis included age at the time of surgery, final pathologic stage (FIGO 2009 staging) [18], histologic subtype, lymphovascular space invasion, body mass index (BMI), uterine mobilizer use, and adjuvant treatment (chemoradiotherapy, chemotherapy and radiotherapy alone). Histology was categorized into low grade (including grade 1 and 2 endometrioid carcinomas) and high grade (including grade 3 endometrioid carcinoma, serous carcinoma, carcinosarcoma, and non-endometrioid subtypes). Both extensive and focal lymphovascular space invasions were classified as positive. If the presence of lymphovascular space invasion was determined to be an artifact secondary to the introduction of the uterine mobilizer, it was documented and categorized as negative.

Covariates included in the multivariable analysis were selected based on established clinical relevance and previously identified associations with recurrence risk in endometrial cancer, rather than data-driven selection methods. Adjuvant treatment variables (chemotherapy, radiotherapy, and chemoradiotherapy) were included to account for their potential confounding effect on recurrence outcomes, as treatment allocation is closely linked to underlying disease risk and may independently influence recurrence-free survival.

Missing data were evaluated for all covariates prior to analysis. Data completeness was high across all variables, with no clinically meaningful missingness identified (<1% across all variables), including stage, histology, LVSI status, and adjuvant treatment variables. Given the negligible extent of missing data and absence of any systematic pattern, a complete case analysis was performed. No imputation methods were applied.

### 2.2. Statistical Analysis

Clinical and pathological characteristics were analyzed using Student’s *t*-test, chi-squared test, or Fisher’s exact test. Survival curves were generated using a Kaplan–Meier model. The multivariate Cox proportional hazards model was used to estimate hazard ratios for the covariates of interest with a 95% confidence interval. The time to event was defined as the time from surgery to the date of diagnosed recurrence. Patients were right-censored if they were lost to follow-up after the last documented clinic visit or if death occurred and was not related to their malignancy. Hazard ratios and confidence intervals were determined using the log-rank test with a two-sided significance level of 0.05. All statistical analyses were performed using R 3.6.0+, which is available online.

The multivariable Cox proportional hazards model included age at procedure, BMI, FIGO stage, histology, LVSI status, uterine manipulator use, and adjuvant treatment indicators (chemotherapy, chemoradiotherapy, and radiotherapy).

The proportional hazards assumption was formally assessed using Schoenfeld residuals with both covariate-specific and global tests. Non-proportionality was defined as evidence that the effect of a covariate on recurrence risk changed over the duration of follow-up, rather than remaining constant over time as assumed by the Cox proportional hazards model.

In accordance with the journal’s guidelines, we will provide our data for independent analysis by a selected team by the Editorial Team for the purposes of additional data analysis or for the reproducibility of this study in other centers if such is requested.

## 3. Results

**Figure 1:**
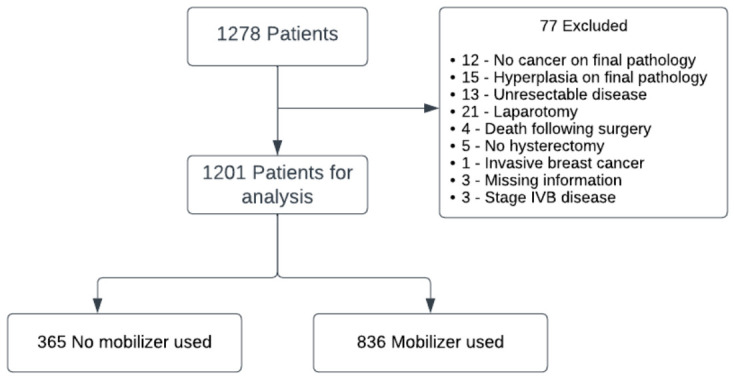
Flow chart of patient analysis.

After reviewing charts of 1278 patients, 77 cases were excluded for final analysis as summarized in Figure 1.

Twelve cases were excluded as there was no evidence of cancer on final pathology, and 15 cases were excluded due to pre-invasive disease on final pathology. Additional cases were excluded as follows: thirteen cases at the time of surgery had evidence of unresectable disease. Four cases involved patients who passed away postoperatively, unrelated to their cancer. Five cases involved patients who did not undergo a hysterectomy at the time of surgery but had staging procedures, including lymphadenectomy and salpingo-oophorectomies. One patient had diffusely metastatic breast cancer, and surgery was for palliative purposes. Finally, three charts were excluded from our final analysis due to missing information.

Of the remaining 1201 patients, 836 patients had uterine mobilizer use at the time of surgery compared to no mobilizer use in 365 patients. In total, 155 cases of recurrent disease were identified. The median duration of follow-up for all patients was 340 days. Characteristics between the two groups of manipulator use are summarized in Table 1.

There was a significant difference in the number of recurrences between the patients with and without mobilizer use at the time of surgery (125 (15.0%) vs. 29 (7.9%), respectively, *p* < 0.001). Known risk factors for recurrence, including stage, histology, and lymphovascular space invasion, did not differ between these two groups. Adjuvant chemoradiotherapy and chemotherapy were significantly different between groups. However, when combining all three treatment modalities, there was no significant difference between the two groups with respect to adjuvant therapy. Age, BMI and adjuvant radiotherapy were not significantly different between groups with and without mobilizer use.

Lymphovascular space invasion was separately categorized if identified as an artifact due to the use of a uterine mobilizer (see Table 2). Thirty-nine cases of lymphovascular space invasion attributed to manipulation within the endometrial cavity were identified; 36 (92.3%) were associated with mobilizer use, compared to 3 (7.7%) without mobilizer use (*p* = 0.003). In the absence of a mobilizer, the artifact was noted to be a processing artifact. Six patients with lymphovascular space invasion due to artifact were identified with recurrence, compared to 33 without evidence of recurrence. There was no significant association between recurrence and the presence of lymphovascular space invasion due to artifact (*p* = 0.81).

**Figure 2:**
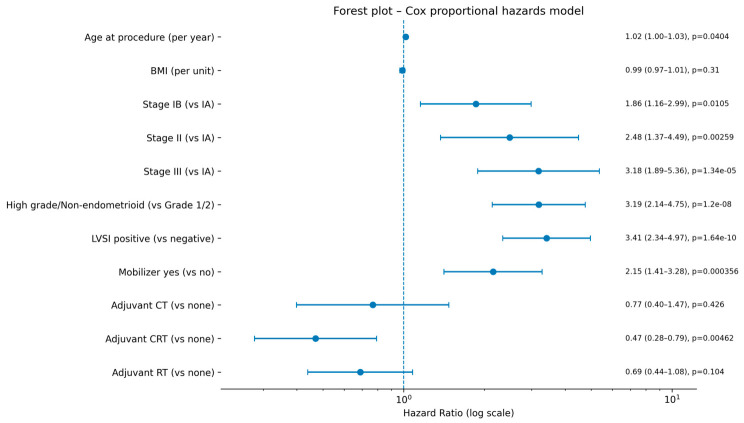
Cox proportional hazards model.

Prognostic factors for tumor recurrence in our multivariate analysis using Cox proportional hazards model are shown in Figure 2. BMI was not significantly associated with a change in recurrence risk. Stages IB, II, and III were associated with an increased risk of recurrence, with hazard ratios (HR) of 1.86, 2.48, and 3.18, respectively (*p* = 0.01, *p* < 0.01, and *p* < 0.01). High-grade endometrioid and non-endometrioid histologies combined as a group were also significant, with an HR of 3.19 (*p* < 0.01). Lymphovascular space invasion demonstrated the greatest risk for recurrence (HR 3.41, *p* < 0.01). Lastly, the multivariate analysis showed that the use of a uterine manipulator was an adverse risk factor (HR 2.15, *p* < 0.01). Chemotherapy and radiotherapy each alone trended towards reduced hazard ratios of 0.77 and 0.69 but neither were statistically significant (*p* = 0.43 and *p* = 0.10). Combined chemoradiotherapy, however, was associated with a reduced hazard ratio of 0.47 (*p* < 0.01).

In a sensitivity analysis restricted to patients who did not receive adjuvant chemoradiotherapy, uterine manipulator use remained significantly associated with recurrence (HR 2.74, *p* < 0.001).

Evidence of non-proportionality was identified for LVSI and adjuvant chemotherapy, while all other covariates satisfied the proportional hazards assumption. The global test indicated modest overall deviation from proportionality. Specifically, this suggests that the magnitude of association between certain covariates and recurrence risk may have varied over the follow-up period, rather than remaining constant over time.

Given that the primary exposure of interest (uterine manipulator use) satisfied the proportional hazards assumption, and that the direction and magnitude of its association were consistent across sensitivity analyses, the model was retained. Alternative modeling approaches, including time-varying coefficient models and stratified Cox analyses, were considered for covariates demonstrating evidence of non-proportionality. However, because the deviation from proportionality was modest overall and did not involve the primary exposure of interest, the standard multivariable Cox proportional hazards model was retained to preserve interpretability and consistency with the prior endometrial cancer literature. Findings are interpreted with appropriate caution, particularly with respect to covariates demonstrating time-varying effects.

**Figure 3:**
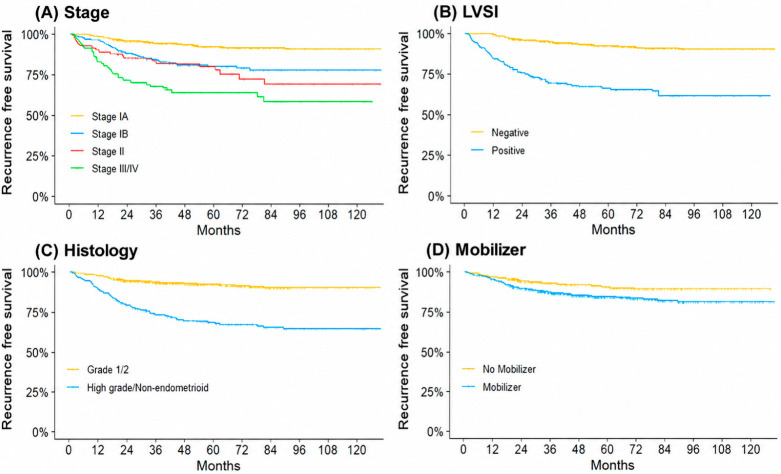
Recurrence-free survival according to (**A**) stage, (**B**) lymphovascular space invasion status, (**C**) histology, and (**D**) uterine manipulator use.

The Kaplan–Meier curves shown in Figure 3 demonstrated recurrence-free survival for patients with stages IA, IB, II, and III to be 91.4%, 77.9%, 69.9%, and 59.0%, respectively. Survival at 10 years was 61.2% for those with lymphvascular space invasion and 90.0% without. Recurrence free survival for those with grade 1 and 2 endometrioid carcinoma at 10 years was 90.5% compared to 64.8% in patients with high-grade endometrioid or non-endometrioid histologies. Survival with and without uterine manipulator use was 81.5% and 89.6% at 10 years respectively.

## 4. Discussion

This study aimed to identify risk factors for endometrial cancer recurrence using a unique cohort of patients who exclusively underwent robotic-assisted laparoscopic hysterectomy. The analysis confirmed that traditional prognostic variables such as age, stage, histology, and lymphovascular space invasion remained significantly associated with recurrence. The presence of lymphovascular space invasion was the most significant predictor of recurrence. High-grade and non-endometrioid histologies were also strongly associated with increased risk. A progressive increase in recurrence risk was observed with advancing stage beyond IA.

A key finding unique to this study was the association between uterine manipulator use and higher risk of recurrence. Additionally, adjuvant chemoradiotherapy was associated with a statistically significant reduction in recurrence, whereas chemotherapy or radiotherapy alone was not. Interaction testing between uterine manipulator use and histologic subtype did not yield a statistically significant association.

The choice of uterine manipulator was based primarily on surgeon preference rather than specific tumor or uterine characteristics. Within the surgical group, most surgeons initially used the V-Care manipulator until it became apparent that one surgeon had been using a vaginal probe, coinciding with the period when the LACC trial data were emerging. Device selection was not influenced by tumor size, uterine size, or case complexity. Following the publication of the cervical cancer data, the group transitioned uniformly to the alternative approach. There was no observed correlation between device selection and operative time or difficulty with uterine retrieval.

However, the association between uterine manipulator use and recurrence should be interpreted as associative rather than causal. Given the retrospective, non-randomized design, selection bias and unmeasured confounding cannot be excluded. Importantly, manipulator use was determined by surgeon preference and practice patterns rather than random allocation. Although baseline characteristics were generally balanced between groups, residual confounding may persist despite adjustment for known prognostic variables and adjuvant treatment factors. Unmeasured variables related to operative decision making, intraoperative tumor handling, or case selection may also have contributed to the observed association.

Our findings corroborate well-established risk factors for endometrial cancer recurrence, including older age, advanced stage, high-grade or non-endometrioid histology, and lymphovascular space invasion [14,19]. These associations have been consistently demonstrated across numerous studies and were reaffirmed in this robotic-only surgical cohort.

The association between uterine manipulator use and recurrence is a key novel contribution. The LACC trial in cervical cancer first raised concerns about minimally invasive surgery, particularly implicating the use of uterine manipulators as a potential contributor to poorer outcomes [20]. Prior studies have suggested that these devices may contribute to iatrogenic tumor dissemination through direct disruption or seeding of malignant cells into the lymphovascular system or peritoneal cavity [21,22,23]. Our data add to this hypothesis, showing a significantly increased hazard of recurrence in patients where manipulators were used, independent of tumor grade or histology.

Although adjuvant chemoradiotherapy was associated with reduced recurrence risk, sensitivity analyses excluding patients who received chemoradiotherapy demonstrated a consistent association between uterine manipulator use and recurrence. The differential use of adjuvant chemoradiotherapy could represent a potential source of confounding. Chemoradiotherapy was included as a covariate in all multivariable Cox proportional hazards models. In addition, we performed a prespecified sensitivity analysis restricted to patients who did not receive adjuvant chemoradiotherapy. In this stratified analysis, uterine manipulator use remained independently associated with increased recurrence risk (HR 2.74, *p* < 0.001), with effect estimates consistent with and slightly stronger than those observed in the primary model. Nonetheless, residual treatment-selection bias cannot be entirely excluded given the retrospective nature of the cohort.

We did not find BMI to be significantly associated with recurrence in our cohort, which contrasts with some previous reports linking higher BMI to worse outcomes, particularly in low-grade endometrioid cancers [24]. This discrepancy may be due to differences in study populations, operative technique, or statistical power. Our interaction analysis between uterine manipulator use and histology did not show a significant association, suggesting that the detrimental effect of manipulators regardless of tumor biology.

Regarding adjuvant therapy, our finding that chemoradiotherapy significantly reduced recurrence aligns with existing data, including the PORTEC-3 trial, which supports combined modality treatment for high-risk patients. However, our inability to show benefit from chemotherapy or radiotherapy alone may reflect limited sample sizes or heterogeneous treatment indications over the years covered in our dataset.

A major strength of our study is the exclusive focus on robotic-assisted laparoscopic hysterectomy, providing a homogeneous surgical population and eliminating variability introduced by mixed surgical approaches. Additionally, our institution’s standardized robotics training requirements enhanced consistency in surgical technique across providers. The inclusion of uterine manipulator use enabled a novel analysis of modifiable surgical factors influencing recurrence.

Another strength is the identification and separate analysis of artifact-related lymphovascular space invasion, a level of granularity not typically captured in retrospective datasets. We acknowledge that the number of cases in which lymphovascular space invasion was attributed to manipulation or specimen processing artifact was small, which limits the ability to draw definitive statistical conclusions. Accordingly, this component of the analysis should be considered exploratory in nature. For the purposes of the multivariable models, cases deemed artifactual were not included in our multivariate analysis.

Although focal lymphovascular space invasion was historically considered of uncertain significance, emerging evidence suggests prognostic relevance [25]. Our binary lymphovascular space invasion classification reflects this evolving understanding and hence included both focal and extensive as positive.

Nonetheless, this study has several limitations. Its retrospective design and single-center setting limit generalizability. Although all surgeons met standardized robotic training requirements, there may still be unmeasured variation in intraoperative decision making. The use of manipulators was not randomized and was based on surgeon discretion, introducing potential selection bias, even though baseline characteristics were balanced between groups. Additionally, certain subgroup analyses, such as the effect of chemotherapy alone may have been underpowered due to small sample sizes. Surgeon-related variability also represents a potential source of bias. Although procedures were performed within a high-volume tertiary center with standardized robotic training requirements, differences in surgical technique, tissue handling, colpotomy approach, or threshold for manipulator use could not be fully captured within the retrospective dataset. Furthermore, the institutional transition away from uterine manipulators occurred in parallel with evolving evidence following publication of the LACC trial, introducing the possibility of temporal practice-related confounding.

The absence of molecular classification and hormonal receptor status represents a major limitation of this study. Molecular classification systems, including POLE-mutated, p53-abnormal, mismatch repair deficient (MMRd), and no specific molecular profile (NSMP) subtypes, are increasingly recognized as key determinants of prognosis and treatment selection in endometrial cancer, and in many cases may supersede traditional clinicopathologic risk factors. The recent literature has further reinforced the prognostic and therapeutic significance of molecular classification in modern endometrial cancer management [26]. Failure to account for this dimension introduces residual confounding that may influence the observed associations. Molecular classification data (POLE-mutated, p53-abnormal, MMR deficient, NSMP) and estrogen receptor status were not routinely available for this historical cohort (2012–2019) and therefore could not be incorporated into the analysis. It is possible that imbalances in underlying molecular subtype distribution between groups may have contributed to differences in recurrence outcomes independent of uterine manipulator use. As such, our findings should be interpreted within the context of a pre-molecular era cohort and warrant validation in future studies incorporating contemporary molecular risk stratification.

Although recurrence-free survival was estimated for up to 10 years, the median duration of follow-up at the tertiary cancer center was 340 days. Following discharge from specialist care, patients continued longitudinal follow-up within the community, allowing recurrence events to be captured beyond the period of active cancer center surveillance. However, the number of patients at risk at later time points was limited, and long-term survival estimates should therefore be interpreted cautiously.

The most impactful contribution of this study is the identification of uterine manipulator use as a potentially modifiable risk factor for endometrial cancer recurrence. While the device facilitates exposure and operative efficiency, its association with increased recurrence risk warrants serious consideration. Avoidance of uterine manipulators, especially in cases of known or suspected malignancy, may reduce the risk of disease dissemination and recurrence, and alternative surgical strategies should be explored. However, these findings should be interpreted cautiously and viewed as hypothesis generating rather than practice changing in isolation, as prospective validation is required before definitive causal conclusions can be established.

These findings should influence surgical decision making in endometrial cancer management, particularly in patients with early-stage disease where recurrence prevention is critical. Future prospective studies or randomized trials are needed to validate the causal relationship between uterine manipulator use and recurrence, assess biological mechanisms of dissemination, and explore mitigation strategies such as protective barriers, no-touch techniques, or alternative uterine handling methods. Advances in endometrial cancer management will likely extend beyond refinement of robotic surgical techniques alone and increasingly incorporate artificial intelligence-based approaches for risk stratification, surgical planning, recurrence prediction, and individualized treatment selection. Emerging work suggests that integration of artificial intelligence with molecular and clinicopathologic data may improve prognostic accuracy and perioperative decision making in gynecologic oncology [27].

We acknowledge as a limitation that this study included only robotic cases. This reflects the practice pattern at our center, where robotic surgery is the standard approach and conventional total laparoscopic hysterectomy is not performed, precluding a direct comparison between surgical modalities.

Additionally, further research should explore the molecular or immunologic consequences of intraoperative tumor disruption and its potential interaction with adjuvant treatment efficacy. Our findings emphasize the importance of ongoing scrutiny of surgical tools and techniques in oncologic outcomes.

## 5. Conclusions

Known risk factors for recurrence are unchanged in a population of patients with exclusive use of robotic surgery. Poorer recurrence-free survival in patients with high-grade or non-endometrioid histologies and lymphovascular space invasion presents a strong case for further escalating adjuvant therapy and increasing surveillance. As the endometrial cancer molecular classification landscape continues to evolve, treatment decisions and clinical management strategies will be further refined. Our study suggests that uterine manipulator use may be associated with an increased risk of recurrence; however, these findings should be considered hypothesis generating and warrant prospective validation before any changes to clinical practice can be recommended.

## Figures and Tables

**Table 1:** Characteristics between patients with and without mobilizer use ^1^.

	Mobilizer		
	No (%) (N = 365)	Yes (%) (N = 836)	*p*-Value
**Age (Mean)**	63.7	62.5	0.08
**BMI (Mean)**	34.2	34.1	0.89
**Recurrence**			<0.001
Yes	29 (7.9)	125 (15.0)	
No	336 (92.1)	711 (85.0)	
**Stage**			0.80
IA	232 (63.6)	521 (62.3)	
IB	71 (19.5)	179 (21.4)	
II	24 (6.6)	59 (7.0)	
IIIA/B/C1/C2	38 (10.4)	77 (9.2)	
**Histology**			0.37
Grade 1/2	285 (78.1)	631 (75.5)	
High grade/Non-endometrioid	80 (21.9)	205 (24.5)	
**Lymphovascular space invasion**			1.00
Positive	86 (23.5)	195 (23.4)	
Negative	279 (76.4)	640 (76.6)	
**Chemoradiotherapy**			0.02
Yes	29 (7.9)	108 (12.9)	
No	336 (92.1)	728 (87.1)	
**Chemotherapy**			0.02
Yes	26 (7.1)	31 (3.7)	
No	339 (92.9)	805 (96.3)	
**Radiotherapy**			0.38
Yes	76 (20.8)	195 (23.3)	
No	289 (79.2)	641 (76.7)	
**Adjuvant Therapy ^2^**			0.18
Yes	131 (35.9)	334 (40.0)	
No	234 (64.1)	502 (60.0)	

^1^ Percentages may not total 100 because of missing data; ^2^ Combination of chemoradiotherapy, chemotherapy alone and radiotherapy alone.

**Table 2:** Association between lymphovascular space invasion, artifact and recurrence.

	Lymphovascular Space Invasion from Artifact		
	Yes	No	*p*-Value
**Mobilizer use**			
Yes	36	801	0.003
No	3	364	
**Recurrence**			
Yes	6	149	0.8142
No	33	1017	

## Data Availability

Our data is located in a secured database available upon request through our location institution.
